# Insect Antimicrobial Peptide Complexes Prevent Resistance Development in Bacteria

**DOI:** 10.1371/journal.pone.0130788

**Published:** 2015-07-15

**Authors:** Sergey Chernysh, Natalia Gordya, Tatyana Suborova

**Affiliations:** 1 Laboratory of Insect Biopharmacology and Immunology, Faculty of Biology, St. Petersburg State University, St. Petersburg, Russia; 2 Research Center of Kirov Military Medical Academy, St. Petersburg, Russia; Institut National de la Recherche Agronomique, FRANCE

## Abstract

In recent decades much attention has been paid to antimicrobial peptides (AMPs) as natural antibiotics, which are presumably protected from resistance development in bacteria. However, experimental evolution studies have revealed prompt resistance increase in bacteria to any individual AMP tested. Here we demonstrate that naturally occurring compounds containing insect AMP complexes have clear advantage over individual peptide and small molecule antibiotics in respect of drug resistance development. As a model we have used the compounds isolated from bacteria challenged maggots of Calliphoridae flies. The compound isolated from blow fly *Calliphora vicina* was found to contain three distinct families of cell membrane disrupting/permeabilizing peptides (defensins, cecropins and diptericins), one family of proline rich peptides and several unknown antimicrobial substances. Resistance changes under long term selective pressure of the compound and reference antibiotics cefotaxime, meropenem and polymyxin B were tested using *Escherichia coli*, *Klebsiella pneumonia* and *Acinetobacter baumannii* clinical strains. All the strains readily developed resistance to the reference antibiotics, while no signs of resistance growth to the compound were registered. Similar results were obtained with the compounds isolated from 3 other fly species. The experiments revealed that natural compounds containing insect AMP complexes, in contrast to individual AMP and small molecule antibiotics, are well protected from resistance development in bacteria. Further progress in the research of natural AMP complexes may provide novel solutions to the drug resistance problem.

## Introduction

The global expansion of antibiotic resistant bacteria is a major threat to human health. Despite great progress in better knowledge of the resistance mechanisms, the solution to this problem remains elusive [1]. Many efforts have been made to employ antimicrobial peptides (AMPs) as anti-infective drugs with protection against resistance development [2–4]. However, the growing body of evidence demonstrates that therapeutic AMPs have no real advantage over conventional antibiotics since bacteria possess many ways to neutralize AMPs through enzymatic degradation, mutation of target structures, decrease of cell membrane permeability, membrane net charge alteration, active extrusion, etc. [5–7]. It is no wonder that bacteria, according to the experimental evolution studies reviewed below, rapidly lose susceptibility towards any individual AMP tested so far. It is quite amazing that natural AMPs could remain antibacterial to the present day.

In our opinion, based on the study of insect immunity, a possible solution to the riddle lies in the fact that the immune system engages not a single AMP but a battery of active molecules integrated into a co-adapted antimicrobial peptide complex [8]. The capacity for preventing resistance development, from that standpoint, is a feature of the complex as a whole, but not individual compounds.

To examine this idea we have selected as a model a semi-purified AMP complex of bacteria-challenged blow fly *Calliphora vicina* (Diptera, Calliphoridae) larvae. *C*. *vicina* as well as many other Calliphoridae flies are synantropic insects living in locations like animal wounds, dead bodies and excretae highly contaminated with human and animal pathogenic microflora [9]. Since animal and human pathogens are obligatory attribute of Calliphoridae flies environment, they must be well adapted to this kind of infection. *C*. *vicina* larvae are known to respond to bacterial infection or septic injury by production and accumulation in the hemolymph of AMP complex comprising all major families of insect AMPs like defensins, cecropins, diptericins, proline-rich peptides and antiviral peptides alloferons [8, 10–11]. The antibacterial activity spectrum of the complex covers different groups of human pathogens from *Enterobacteriacea*, *Coccaceae*, *Enterococcaceae*, *Pseudomonadaceae*, *Moraxellaceae* and *Corynebacteriaceae* families commonly present in the larvae natural habitats [8].

Antibiotic multi-resistant clinical strains of *Escherichia coli*, *Klebsiella pneumoniae* and *Acinetobacter baumannii* sensitive to the complex antibacterial activity [12] have been employed in antimicrobial selective pressure experiments as model species. Recent efforts to combat these Gram-negative bacteria have come into particular prominence with regard to the antibiotic resistance problem. Carbapenem and third-generation cephalosporin resistant strains of *E*. *coli* and *K*. *pneumoniae* are recognized as the most urgent threats to human health worldwide [13]. *A*. *baumannii* is also in the list of the most dangerous pathogens resistant to all or nearly all antibiotics [14].

The overall aim of this study was to elucidate a difference in resistance development under selective pressure of the natural compounds containing insect AMP complexes and conventional antibiotics. Based on the results of antimicrobial selective pressure experiments, we propose a novel approach to the prevention of drug resistance development in bacterial pathogens. Moreover, we suggest use of *C*. *vicina* naturally occurring AMP complex as a drug candidate effective against *E*. *coli*, *K*. *pneumoniae* and *A*. *baumannii* antibiotic multiresistant strains.

## Materials and Methods

### Insects

Insect species used in experiments were obtained from the Laboratory of Insect Biopharmacology and Immunology of the St. Petersburg State University. Experiments were performed with a wild type laboratory strain of *C*. *vicina* characterized by stable larval diapause [15, 16]. Breeding conditions were essentially the same as previously described [10]. To induce diapause in the progeny, adult flies were kept under short day conditions (12L:12D). The larvae were fed by fresh beef in not sterile conditions at 12°C, III instar larvae were transferred to 3°C at the end of feeding period, left there for 2 weeks to form diapause and then taken to the experiments. In addition to *C*. *vicina* we have used three other dipteran species: blue blow fly *Calliphora vomitoria* belonging to the same genus, green bottleneck fly *Lucilia sericata* from the same Calliphoridae family, and house fly *Musca domestica* from the evolutionary distant Muscidae family. Diapausing III instar larvae of *C*. *vomitoria* and *L*. *sericata* were obtained in accordance with the same protocol as *C*. *vicina*. III instar larvae of *M*. *domestica* having no diapause in their life cycle were maintained at constant temperature 25°C and used in experiments shortly after the end of the feeding period.

### Preparation of natural compound containing *C*. *vicina* AMP complex

Mixture of *Escherichia coli* D31 and *Micrococcus luteus* A270 cells have been used to induce immune response in diapausing *C*. *vicina* larvae. One-day cell cultures were grown on the surface of solid LB agar nutritive medium in sterile conditions, individual colonies were picked up, transferred into flasks with 200 mL of liquid nutritive agar medium (Luria Broth Base, 25 g/L) and incubated overnight at 37°C. Then bacterial cells concentration was calculated using the suspension optical density measurement and the cells were sedimented by centrifugation (tabletop centrifuge, 3000g, 15 min). Then the supernatant was removed and the cells were resuspended in the nutritive medium to adjust their concentration to 10^11^ cells/mL. Finally, *E*. *coli* and *M*. *luteus* suspensions were pooled in a 1:1 ratio. The larvae were pricked with a needle previously dipped into the suspension and were left overnight at 25°C. Their surface was then sterilized in 70% ethanol, washed with distilled water and dried. Hemolymph (approximately 10 μl per animal) was collected in ice-cold tubes through a cuticle puncture. Hemolymph samples were kept at -70°C until use. Thawed hemolymph was acidified with 0.1% trifluoroacetic acid (TFA) to a final concentration of 0.05% and insoluble particles were removed by centrifugation (30 min at 8000g at 4°C). The supernatant was applied to reversed-phase Sep-Pak C18 cartridges (Waters) stabilized by 0.05% TFA in the amount of 5 mL/g of sorbent. Highly hydrophilic compounds were removed by cartridge washing with 0.05% TFA. Compounds absorbed in the cartridge were eluted with 50% acetonitrile solution acidified with 0.05% TFA, lyophilized (FreeZone, Labconco) and stored at -70°C. Prior to use, the lyophilized sample was dissolved in deionized water (50 mg/mL), sterilized by filtration through a membrane with a pore size 0.22 μm (Milliex-GS, Millipore) and frozen at -70°C.

### *C*. *vicina* AMP complex characterization

Natural compound containing *C*. *vicina* AMP complex was characterized by a combination of reversed phase HPLC, MS and bacterial growth inhibition assays. 1 mg of the lyophilized compound was dissolved in deionized water and applied to Shimadzu LC20 Prominence HPLC system equipped with analytical column C18 Vydac (4.6 х 250 mm, 5 μm, Grace), equilibrated with 0.05% TFA. The column was eluted with a linear gradient of acetonitrile (ACN) from 0 to 50% in acidified water (0.05% TFA) for 50 min [17]. Chromatographic fractions were automatically collected with 1 min intervals. The fractions’ optical densities were registered by means of a UV detector at two fixed wavelengths 214 and 280 nm. The fractions were lyophilized, dissolved in deionized water and tested against *M*. *luteus* A270 and *E*. *coli* D31 using the plate growth inhibition assay described below. Active antibacterial fractions were analyzed by MS (MicroTOF ESI, Bruker Daltonics) and experimentally determined masses were compared with the previously published characteristics of *C*. *vicina* individual AMPs [8, 10]. The peptides were sequenced by Edman degradation method as described [10].

### Bacteria

*Escherichia coli* D31 and *Micrococcus luteus* A270 strains routinely used in insect AMP studies were obtained from the Institute of Genetics and Molecular and Cellular Biology (IGBMC) [17]. Clinical strains of *E*. *coli*, *Klebsiella pneumoniae* and *Acinetobacter baumannii* used in the antibiotic/antimicrobial selective pressure experiments were obtained from infected patients of the surgery clinic of the Kirov Military Medical Academy (St. Petersburg, Russia). Profiles of the strains’ antibiotic resistance were determined as recommended (National Committee for Clinical Laboratory Standards, 2003. Performance Standards for Antimicrobial Disk Susceptibility Tests. Approved standard M2-A8. NCCLS, Wayne, PA). The strains were classified as susceptible (S), intermediate (I) or resistant (R) to the antibiotic tested by disc diffusion method (Table 1).

**Table 1:** Antibiotic resistance spectra of bacterial strains used in selection experiments.

Strain / Antibiotic	Amc	Ami	Net	Gen	Ipm	Mem	Chl	Cip	Cfp	Cfp+Sul	Caz	Ctx	Cpe
*E*. *coli* 774.1	S	S	nd*	S	S	S	S	S	S	S	nd*	S	nd*
*E*. *coli* 863.1	R	R	R	R	S	S	I	R	R	S	R	R	I
*K*. *pneumoniae* 104.2	I	R	R	R	S	S	I	R	R	R	R	R	R
*A*. *baumanii* 82.2	R	R	R	R	R	R	R	I	R	S	R	R	R

Antibiotic abbreviations: Amc—amoxicillin/clavulanic acid, Ami–amikacin, Net–netilmicin, Gen–gentamicin, Ipm–imipenem, Mem–meropenem, Chl–chloramphenicol, Cip–ciprofloxacin, Cfp—cefoperazone, Cfp/sul–cefoperazone, Sul—sulbactam, Caz–ceftazidime, Ctx–cefotaxime, Cpe–cefepime.

*- no data.

### Antimicrobials

The compound containing *C*. *vicina* AMP complex was prepared for selection experiments and characterized in accordance with the protocols described above. For comparison, similar compounds were isolated from three other insect species: *C*. *vomitoria*, *L*. *sericata* and *M*. *domestica*. Protocols of the compounds preparation were essentially the same as described for *C*. *vicina* compound. MICs for each preparation were determined before selection experiments using the microdilution method described below.

Third generation cephalosporin cefotaxime, meropenem from carbopenems’ group and polypeptide polymyxin B were applied as reference antibiotics in the selection experiments. These antibiotics were chosen because of their clinical importance and relevance to the bacteria tested. Carbapenems and third generation cephalosporins are the most important antibiotics for the treatment of *E*.*coli* and *K*. *pneumonia* infections however their therapeutic efficacy is dramatically decreased by growing prevalence of beta-lactamase producing strains [13]. Polymyxin B is an antibiotic primarily used for resistant Gram-negative infections like beta-lactam resistant *E*.*coli* and *K*. *pneumonia* and multidrug-resistant *A*. *baumannii* [14]. A number of resistance mechanisms to many classes of antibiotics are known to exist in *A*. *baumannii*, including beta-lactamases, efflux pumps, aminoglycoside-modifying enzymes, permeability defects, and the alteration of target sites [18, 19].

The following commercial preparations were used as reference antibiotics in the experiments: sodium cefotaxime (ABOLmed, Duckacha str., No. 4, Novosibirsk, 630096, Russia), meropenem trihydrate (AstraZeneca) and naturally occurring polypeptide polymyxin B sulfate (Kievmedpreparat, Saksaganskogo str., No. 139, Kiev, 01033, Ukraine). The antibiotics were dissolved in sterile deionized water in concentration 1 mg/mL, aliquoted in 0.05 mL volumes and kept at -70°C until use.

### Antibacterial activity assays

Standard plate-growth inhibition assay was employed for identification and relative quantification of the complex active compounds. The method was essentially the same as the one previously described [10]. *E*. *coli* D31 and *M*. *luteus* А270 cultures were grown in LB liquid nutrient medium (Invitrogen) for 18–20 hours at 37°C. Sterile Petri dishes (9 cm in diameter) were filled with 7.5 mL of LB medium supplemented with 12g/L agarose (Invitrogen). 4 x 10^6^ CFU/dish test microorganisms measured by OD were inoculated into the warm medium. The analytes (fractions 1–53 of Table 2) were dissolved in 20 μl of deionized sterile water and 2 μl aliquot of the solution was applied onto a solid medium surface. The diameter of the growth inhibition zone was measured after 24-hour incubation at 37°C and the inhibition zone area was calculated and used for relative quantification of the AMP anti-*M*. *luteus* and anti-*E*. *coli* activity.

**Table 2:** Antibacterial activity, chromatographic, mass spectrometric and structural characteristics of active AMPs present in *C*. *vicina* AMP complex.

HPLC fraction №	Growth inhibition zone, mm^2^		Molecular masses found in the sample, Da	Known AMPs characteristics (Chernysh, Gordja, 2011)		Peptide family
	M. luteus	E. coli		Molecular masses, Da	AA sequence	
0–23	0	0				
24	0	50				
25	0	78				
26	20	95				
27	283	20	4032.6	4032.0	ATCDLLSGTGANHSACAAHCLLRGNRGGYCNGKAVCVCRN	Defensin
28	254	20	4032.6	4032.0		Defensin
			2987.0	2987.0		Proline-rich peptide
29	95	38	2987.0	2987.0	FVDRNRIPRSNNGPKIPIISNP. . .(N-terminus)	Proline-rich peptide
30	50	95	8886.5	8886.2	DSKPLNLVLPKEEPP	Diptericins
			8999.4	8999.7	NNPQTYGGGGGSRKDDFDVVLQGAQEV. . .(N-terminus)	
31	38	177	8886.5			Diptericins
			8914.0	8913.9		
			9029.0	9029.1		
32	20	133				
33	20	78	4156.0	4156.0	GWLKKIGKKIGRVGQHTRDATIQGLAVAQQAANVAATAR	Cecropin
34	0	64	4156.0			Cecropin
35	0	50				
36	0	28				
37	0	24				
38	0	20				
39	0	20				
40	0	16				
41	0	7				
42	0	7				
43	0	7				
44–53	0	0				

The standard microdilution method was carried out for MIC determination with LB broth (Invitrogen), as recommended (National Committee for Clinical Laboratory Standards, 1997. Methods for dilution antimicrobial susceptibility test for bacteria that grow aerobically. Approved standard M7-A4. NCCLS, Wayne, PA).

### Selection experiments

Individual wells of a 96-well tissue culture plate (Sarstedt AG & Co., Newton, NC) containing 100 μl of liquid nutrient medium LB (Invitrogen) with doubling antibiotic dilutions were inoculated with approximately 5 x 10^5^ CFU/mL of test bacteria at antibiotic concentrations ranging from 3 doubling dilutions above to 3 doubling dilutions below the MIC of each agent for each strain. The initial inoculum was grown on the solid LB agar nutritive medium (Invitrogen), individual colonies were picked up, transferred into liquid medium (Luria broth base, 25 g/L) and incubated overnight at 37°C. The plates were incubated at 35°C for 24 hours. For each subsequent daily transfer, 1 μl inoculum was taken from the first well containing a sub-inhibitory drug concentration and sub-cultured into the next passage wells containing each diluted drug. The number of transfers in the presence of antibiotic varied from 15 to 35 depending on the MIC changes monitored in the course of each experiment. Typically, the experiment finished when the MIC value in the control antibiotic treated population reached plateau and remained unchanged during next transfers whereas no changes in the AMP complex treated population were registered. The experiment was continued over the next 15 transfers if MIC value of AMP complex demonstrated a small variability in the course of selection. MICs of the preparation and reference antibiotics were tested in three independent repetitions before and after selection. MIC value was also monitored after each transfer.

### Statistics

MICs before and after selection were measured in three repetitions and analyzed by ANOVA. MIC raw data were transformed into log10 MIC to approximate a normal distribution prior to statistical analysis as recommended [20, 21]. MICs after each transfer were measured in one repetition, numbers of paired timing points varied from 15 to 35 depending on the transfer numbers. The statistical significance of MIC changes in reference antibiotic and *C*. *vicina* AMP complex treated bacteria was evaluated by means of a nonparametric Wilcoxon paired difference test and paired measures ANOVA test. The methods applied for each experiment statistical analysis are specified in relevant places of the Results section. Calculations were made by means of the Primer of Biostatistics software, version 4.03.

## Results

### *C*. *vicina* AMP complex characterization

**Fig 1:**
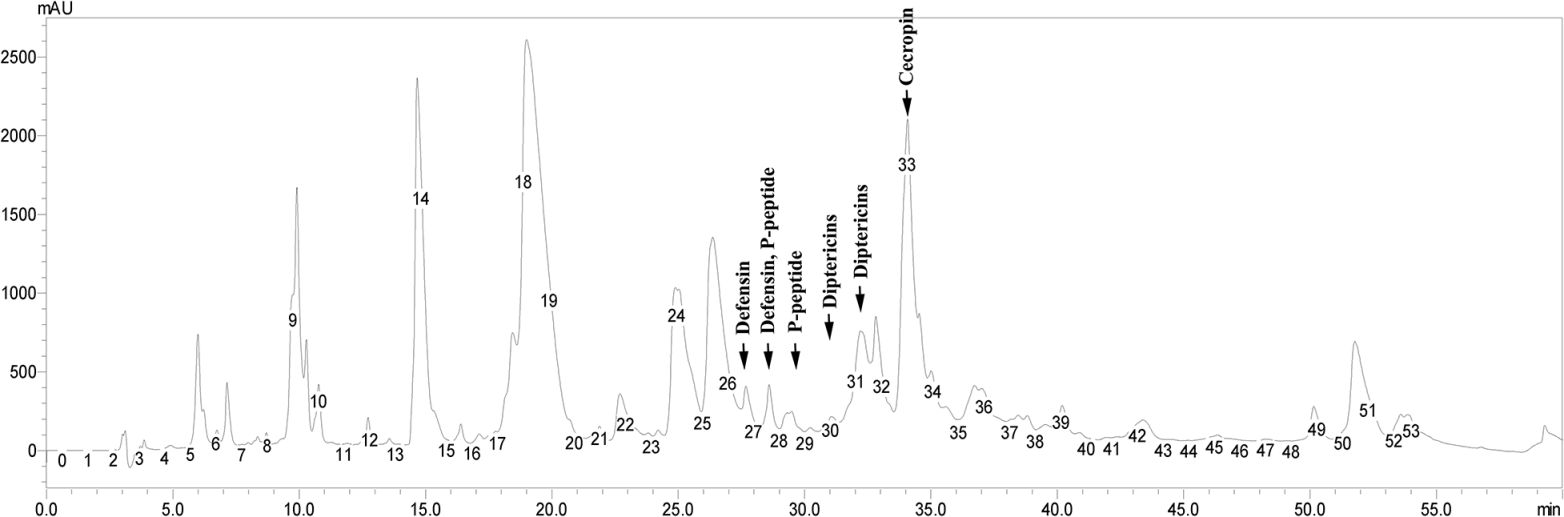
Chromatographic characteristics of naturally occurring compound containing *C*. *vicina* AMP complex. 1 mg of purified complex isolated from bacteria challenged *C*. *vicina* larvae were subjected to reversed-phase HPLC fractionation with 1 min intervals as described in Materials and Methods section. Optical density of the fractions was measured in mAU units at 214 nm wave length. 53 fractions were individually collected, lyophilized and stored at -70°C until further antimicrobial activity and mass spectrometry analyses summarized in Table 2.

To characterize the composition of antimicrobial compounds, 1 mg was fractionated by HPLC (Fig 1). 53 fractions collected with 1 min intervals were lyophilized and their antibacterial activities were quantified through plate growth inhibition assay using Gram-negative *E*. *coli* D31 and Gram-positive *M*. *luteus* A270 bacteria as test-organisms (Table 2). The majority of anti-*M*. *luteus* activity was present in fractions 27 to 30, whereas compounds active against *E*. *coli* were found in a broad range of fractions starting from 24 to 43. MS analysis of fractions 27 to 33 revealed masses precisely corresponding to the masses of defensin, P-peptide, 4 diptericins and cecropin previously isolated from *C*. *vicina* and structurally characterized [8, 10]. Profiles of the peptides’ antibacterial activity (prevalence of anti-*M*. *luteus* or anti-*E*. *coli* activity) and chromatographic mobility were also consistent with known characteristics of the peptides. Relative quantification of the peptides’ activity based on the growth inhibition zone calculation (Table 2) shows that defensin is a leading anti-*M*. *luteus* constituent while proline-rich peptide seems to take second place. Diptericins are responsible for the most part of the complex anti-*E*. *coli* activity complemented by cecropin and a series of unidentified compounds. Summarizing the data of MS, chromatography and bioassays, we conclude that the complex contains four families of insect AMPs known in *C*. *vicina*: defensins, cecropins, diptericins and proline-rich peptides. Moreover, the data obtained demonstrate the presence of some additional antimicrobial substances in the complex, which remain to be structurally characterized, and compounds having no direct antimicrobial activity (Fig 1 and Table 2).

### Resistance development to reference antibiotics and the compound containing C. vicina A MP complex

**Fig 2:**
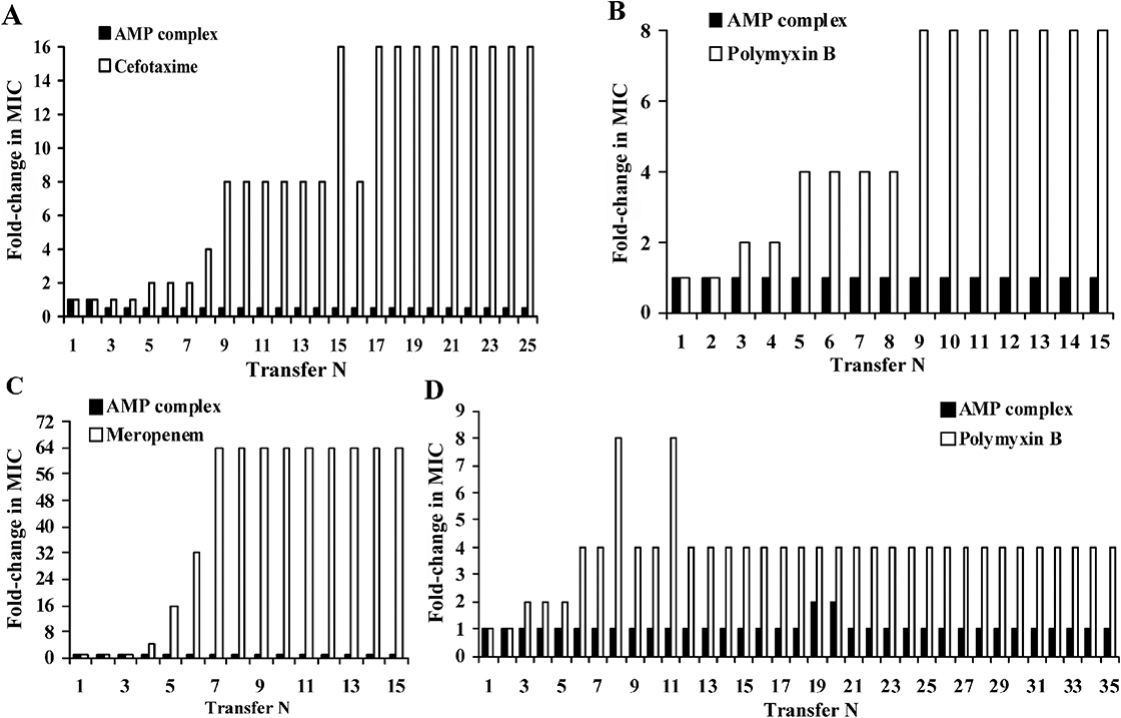
MIC changes in bacterial strains exposed to selection by the compounds containing *C*. *vicina* AMP complex or conventional antibiotics. (A) *E*. *coli* 774.1 (reference antibiotic cefotaxime). *E*. *coli* antibiotic sensitive strain 774.1 was exposed to selection by the AMP complex or cefotaxime in the course of 25 daily transfers as explained in Materials and Methods section. Resistance rate is expressed as fold change in MICs. 1 MIC unit is equal to the MIC value at transfer 1 (250 mg/L for the compound and 0.125 mg/L for cefotaxime, correspondingly). Selection by cefotaxime caused 16-fold increase of MIC while no signs of MIC change were found in the compound treated population. Difference in the compound versus cefotaxime effects on the resistance development was highly significant according to Wilcoxon test statistics (W = 276, n = 23, P<0.001). (B) *E*. *coli* 774.1 (reference antibiotic polymyxin B). The strain was exposed to selection by the compound or polymyxin B in the course of 15 daily transfers. 1 MIC unit is equal to the MIC value at transfer 1 (250 mg/L for the compound and 8.0 mg/L for polymyxin B, correspondingly). Difference in the compound versus polymyxin B effects on the resistance development was highly significant according to Wilcoxon test statistics (W = 91, n = 13, P<0.022). (C) *E*. *coli* 863.1 (reference antibiotic meropenem). *E*. *coli* antibiotic multiresistant meropenem sensitive strain 863.1 was exposed to selection by the compound or meropenem in the course of 15 daily transfers. 1 MIC unit is equal to the MIC value at transfer 1 (500 mg/L for the compound and 0.125 mg/L for meropenem, correspondingly). Difference in the compound versus meropenem effects on the resistance development was highly significant according to Wilcoxon test statistics (W = 78, n = 12, P<0.020). (D) A. baumannii 882.2 (reference antibiotic polymyxin B). *A*. *baumannii* antibiotic multiresistant strain 882.2 was exposed to selection by the AMP complex or polymyxin B in the course of 35 daily transfers. 1 MIC unit is equal to the MIC value at transfer 1 (500 mg/L for the compound and 2 mg/L for polymyxin B, correspondingly). Difference in the compound versus polymyxin B effects on the resistance development were highly significant according to Wilcoxon test statistics (W = 561, n = 33, P<0.001).

Four clinical strains were used in antibiotic/antimicrobial selective pressure experiments: antibiotic sensitive *E*. *coli* 774.1, antibiotic resistant *E*. *coli* 863.1, *K*. *pneumoniae* 104.2 and *A*. *baumannii* 882.2. Experiments with *E*. *coli* 774.1 strain were repeated twice, using cefotaxime (Fig 2A) and polymyxin B (Fig 2B) as reference antibiotics. 16-fold and 8-fold increases in MIC values were registered in bacteria subjected to selection by cefotaxime and polymyxin B, correspondingly. Detectable MIC changes became visible after the first 3 to 5 transfers. MIC increase reached maximum value after the 9-th and 17-th transfers in polymyxin B and cefotaxime treated populations, correspondingly. Subsequently, MICs remained at the maximum level until the end of the experiments. In contrast to the reference antibiotics, MIC of *C*. *vicina* compound demonstrated no changes in the course of the experiments.

The antibiotic multi-resistant meropenem sensitive *E*. *coli* 863.1 strain demonstrated essentially the same results: rapid growth of meropenem resistance up to 64-fold level under selective pressure from the antibiotic and no detectable MIC changes in the compound treated population (Fig 2C).

The antibiotic multi-resistant meropenem sensitive *K*. *pneumoniae* 104.2 strain demonstrated a similar response to selective pressure from the antibiotic and the complex: dramatic 128-fold MIC increase in the meropenem treated population and no MIC changes in the compound treated bacteria as we have reported previously [8].

Polymyxin B resistance development in the *A*. *baumannii* strain 882.2 was limited to stable 4-fold growth while no regular MIC changes were found in the population experiencing the compound repeated treatments (Fig 2D). It is notable that differences between MIC changes in antibiotic and the complex treated populations were highly significant in all experiments according to Wilcoxon test for paired samples as indicated in the figures’ footnotes.

Data characterizing the compound and reference antibiotics MICs before and after selection are summarized in Table 3. Results were essentially the same as the results of the MIC monitoring described above. Selective pressure of reference antibiotics caused statistically significant increase of MIC values (P equal or below 0.001, according to ANOVA test). Maximum MIC values varied from 3- to 128-fold depending on the antibiotic and bacterial strain. At the same time, no statistically significant changes in the compound MICs before and after selection were registered. An analysis of summary data covering all five experiments also found no significant differences in the complex MICs before and after selection (ANOVA, Wilcoxon test).

**Table 3:** Resistance development under selective pressure of cefotaxime, meropenem, polymyxin B and *C*. *vicina* AMP complex.

Selection agent	Transfers number	MIC, mg/L		К_R_ *	P (ANOVA)
		Before selection	After selection		
*E*. *coli* 774.1					
AMP complex	25	250±0.0	330±80	1.32	0.374
Cefotaxime	25	0.17±0.04	2.0±0.0	11.8	<0.001
*E*. *coli* 774.1					
AMP complex	15	250±0.0	420±80.0	1.68	0.116
Polymyxin B	15	6.67±1.33	53.3±10.6	7.99	0.003
*E*. *coli* 863.1					
AMP complex	15	420±80	500±0.0	1.19	0.374
Meropenem	15	0.125±0.0	8.0±0.0	64	<0.001
*K*. *pneumoniae* 104.2					
AMP complex	25	830±170	500±0.0	0.60	0.116
Meropenem	25	0.125±0.0	16.0±0.0	128	<0.001
*A*. *baumannii* 882.2					
AMP complex	35	420±0.08	500±0.0	1.19	0.374
Polymyxin B	35	2.7±0.3	8.0±0.0	2.96	0.007

*K_R_−ratio of MIC after selection to MIC before selection.

Properties similar to *C*. *vicina* compound were demonstrated in the compounds obtainable from other insect species: *C*. *vomitoria*, *L*. *sericata* and *M*. *domestica* (Table 4). Selective pressure of the compounds in the course of 25 consecutive transfers equal to 150–175 generations of *E*. *coli* did not cause statistically significant changes in MIC values.

**Table 4:** Resistance before and after selection by *Calliphora vomitoria*, *Lucilia sericata* and *Musca domestica* AMP complexes in *E. coli* 774.1 strain.

Source of AMP complex	MIC, mg/L		К_R_ *	P (ANOVA)
	Before selection	After selection		
*C*. *vomitoria*	420±80	500±0	1.19	>0.1
*L*. *sericata*	420±80	500±0	1.19	>0.1
*M*. *domestica*	2000±0	3300±700	1.65	>0.1

*KR–ratio of MIC after selection to MIC before selection

**Fig 3:**
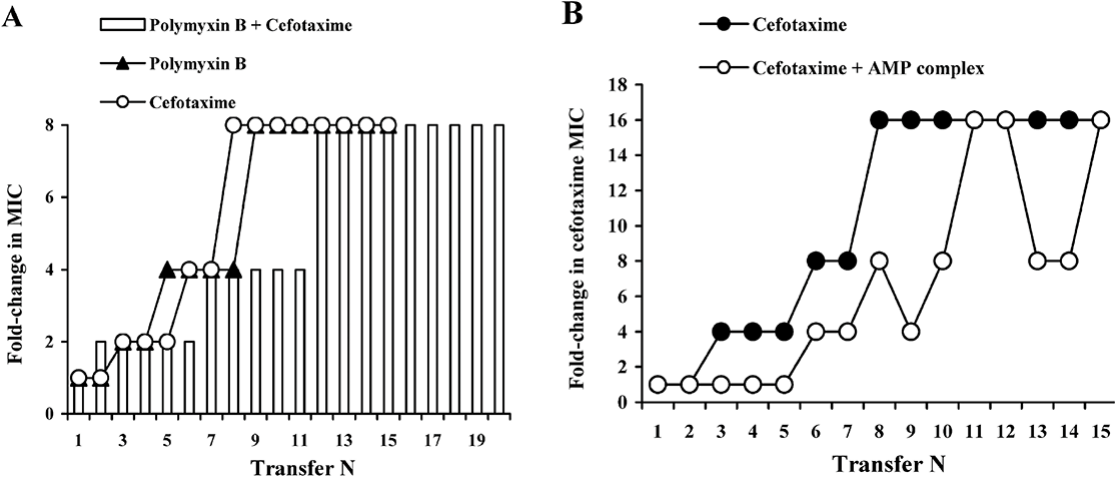
MIC changes in the course of selection by the combinations of antimicrobial agents. (A) Cefotaxime and polymyxin B combination. *E*. *coli* antibiotic sensitive strain 774.1 was exposed to selection by cefotaxime, polymyxin B or a mixture of polymyxin B and cefotaxime in the course of 15–20 daily transfers. Resistance level is expressed as fold change in MICs. 1 MIC unit is equal to 8 mg/L for polymyxin B, 0.125 mg/L for cefotaxime and 1.0 mg/L for a mixture containing cefotaxime and polymyxin B in ratio 1:32, correspondingly. Selection by cefotaxime, polymyxin B or a mixture of the antibiotics caused identical 8-fold increase of MIC. Differences in the mixture versus cefotaxime (W = 19, n = 6, P = 0.062) and polymyxin B (W = 19, n = 6, P = 0.062) effects on the rate of resistance development were statistically insignificant according to Wilcoxon test. (B) The compound containing *C*. *vicina* AMP complex and cefotaxime combination. *E*. *coli* strain 774.1 was exposed to selection by cefotaxime alone or cefotaxime in combination with the compound (50 mg/L) in the course of 15 daily transfers. Resistance level is expressed as cefotaxime fold change in MICs. 1 MIC unit corresponds to MIC value of cefotaxime at transfer 1 (0.125 mg/L). Delay of cefotaxime resistance development in presence of the compound sub-inhibitory concentration was statistically significant according to Wilcoxon test (W = 78, n = 12, P<0.02) and repeated measures ANOVA test (F = 16.465, η = 29, P = 0.001).

Additionally, we compared rates of resistance development to cefotaxime, polymyxin B and a combination of antibiotics in *E*. *coli 774*.*1* antibiotic sensitive strain (Fig 3A). Antibiotics applied one at a time and in a combination caused similar growth of the selection agents’ MICs. Thus, combining two conventional antibiotics did not prevent resistance development in contrast to the insect AMP complexes.

Moreover, results of *C*. *vicina* compound combination with cefotaxime have been analyzed in the same model system (Fig 3B). The complex sub-inhibitory concentration could not prevent cefotaxime resistance development though it distinctly delayed the process.

## Discussion

Animal AMPs combine many favorable properties as potential antimicrobial drugs [2–4]. However, experimental evolution studies revealed prompt resistance growth to any individual AMP tested so far. *Pseudomonas aeruginosa* became resistant to cecropin P1, indolicidin, magainin II, nisin or ranalexin after seven daily cycles of exposure (168 total hours) [22]. Melittin and gramicidin D resistant clones of *Mycoplasma pulmonis* were obtained in two rounds of selection [23]. Seven overnight passages with pexiganan caused MIC increase in 6 of 7 bacterial species tested [24]. The increase was not considered by the authors as noteworthy, nonetheless further experiments with *P*. *aeruginosa* and *E*. *coli* confirmed that pexiganan selects for sharp MIC increase after longer exposure [25]. Resistance development to pexiganan, melittin and iseganan was registered in *Staphylococcus aureus* after two weeks of exposure [26]. Cross-resistance development to host AMPs under selective pressure of therapeutic AMP is particularly alarming [27–29]. It is notable that not only animal AMPs, but also their microbial counterparts like colistin may induce cross-resistance to the host AMPs [29]. From this perspective, the AMP-based platform of antimicrobial drug discovery is debatable now. Taking into consideration the lingering crisis in small molecule antibiotics discovery, it makes the future of antibacterial chemotherapy especially worrisome [1, 30].

Here we tested the capacity of resistance development towards naturally occurring compounds containing insect AMP complexes in comparison with reference antibiotics: polypeptide polymyxin B and beta-lactam antibiotics cefotaxime and meropenem. Eight independent selection experiments with four clinical strains of *E*. *coli*, *K*. *pneumoniae* and *A*. *baumannii* clearly demonstrated that the bacteria readily developed resistance to any individual antibiotic tested. The first signs of the resistance growth were seen after 3 to 5 daily transfers. Taking into account that one daily transfer in similar conditions covers 6 to 7 bacterial generations [25], the growth in our experiments became evident after 18 to 35 generations continuously affected by the antibiotics. It is notable that these data do not allow discriminating genetic (selection of resistant mutants, horizontal transfer of mobile genetic elements) and epigenetic (increased expression of factors that aid resistance) mechanisms of antibiotic resistance development.

Selection with the compound containing *C*. *vicina* AMP complex had quite different consequences. Five independent selection experiments comprising 15 to 35 daily transfers (90 to 245 generations, correspondingly) demonstrated remarkable stability of the complex sensitivity rates in all bacterial strains tested. That strongly distinguishes the compound from conventional antibiotics including therapeutic AMPs. Similar results were obtained in experiments with similar compounds containing AMP complexes of other insects *C*. *vomitoria*, *L*. *sericata and M*. *domestica*. Although none of the bacterial strains was able to acquire resistance against the compounds in our experimental conditions, one cannot exclude that some bacteria may evolve in this way in long-term perspective by means of enhanced production of proteases or another mechanism neutralizing AMPs activity.

Studies of naturally occurring AMPs are mainly focused on the discovery and mode of action analysis of individual peptides, and a little research is dedicated to their interplay in the killing of bacteria [31]. The experiments described in this paper prove for the first time the compounds containing animal AMPs in their naturally occurring combination disable resistance development in bacteria. The mechanism of this phenomenon needs further elucidation including the role of individual major AMPs already characterized, as well as characterization of the structure and functions of other entities present in the compound. Nonetheless, the authors express their willingness to discuss here some ideas based on the available knowledge. The simplest mechanistic explanation is that the simultaneous action of several agents, affecting different targets in bacterial cells minimizes the probability of preexistence of adequate mutations in the population. From that point of view, the multiplicity of AMPs could be sufficient for resistance delay or prevention. To verify the hypothesis, we conducted two additional selection experiments. Firstly, an antibiotic sensitive *E*. *coli* 774.1 strain was exposed to selection by cefotaxime and polymyxin B, applied individually or combined. The rate of the resistance development was about the same in all three experimental groups. Thus, the artificial combination of two different agents did not cause evident delay in the adaptation process in bacteria. In the next experiment cefotaxime MIC changes were measured in the course of selection by the antibiotic alone or in combination with *C*. *vicina* compound. Although the sub-inhibitory concentration of the compound reliably delayed cefotaxime resistance growth, it was not able to restrain it for a long time. Similarly, a combination of two AMPs originating from evolutionary distant organisms, pexiganan from amphibians and mellitin from honey bee venom demonstrated slightly decreased resistance growth in *S*. *aureus* as compared to the individual constituents but was not able to block it [26]. Thus, the multiplicity of antimicrobials alone seems to be insufficient for resistance prevention although it may potentially be useful for expanding the life span of conventional antibiotics.

Furthermore, the phenomenon may hypothetically be attributed to specific features of the individual AMPs constituting the complex. Peschel and Sahl suggested several mechanisms that may help cationic antimicrobial peptides to maintain their functionality during host-pathogen co-evolution [5]. Particularly, the suggestion is illustrated by the “smart” lantibiotic nisin combining five different antimicrobial activities in one molecule [5]. However, selection experiments and the growing prevalence of nisin resistant strains in nature demonstrated that even such a “smart” molecule cannot prevent resistance acquisition [22, 32]. The publications referenced above and the experiments with polymyxin B described here confirm that resistance development is a general rule for any individually applied AMP.

We suggest that both the multiplicity of AMPs and the specific mechanisms of action of the complex constituents are equally important for resistance prevention. *C*. *vicina* AMP complex comprises four major cationic AMP families, which kill bacteria directly: defensins, cecropins, diptericins and proline-rich peptides. *Calliphora* defensin, as well as defensins of other insects and vertebrates, is a peptide with a 3D structure containing α-helix/β-sheet elements coordinated by 3 disulfide bridges and is predominantly active against Gram-positive bacteria. All defensins cause bacterial cell wall disruption/permeabilization although inhibition of the cell wall biosynthesis was demonstrated as well [33, 34]. *Calliphora* cecropin is a linear amphipathic α-helical peptide particularly active towards Gram-negative bacteria. All insect cecropins are known to have pore-forming and cell membrane permeabilizing activity [33]. *Calliphora* diptericins are members of a glycine-rich AMP family selectively toxic to some Gram-negative Enterobacteria like *E*. *coli* by means of cell wall disruption [35]. *Calliphora* proline-rich peptides belong to the family of proline/arginine-rich AMPs. In contrast to defensins, cecropins and diptericins, proline/arginine-rich AMPs are known to kill bacteria by damaging DNA and/or protein synthesis and, therefore, must penetrate inside the target cell [36]. Thus, the *C*. *vicina* AMP complex comprises three structurally distinct groups of cell wall disrupting AMPs targeted predominantly to the membranes of Gram-negative (cecropins, diptericins) or Gram-positive (defensins) bacteria and one group affecting intracellular targets (proline-rich peptides). Since the activity of intracellularly targeted toxin is inevitably dependent of penetration through the cell membrane, a synergy of the complex proline-rich peptides and membrane permeabilizing constituents looks quite plausible. A synergy of proline-rich AMP and defensins has been confirmed with the example of the oyster *Crassostrea gigas* antimicrobials [37]. Differences in structure, antibacterial activity spectrum and toxicity mechanisms of defensins, cecropins and diptericins also prerequisite their synergetic or at least additive interaction. It is possible that combination of these four peptide families was formed in the course of flesh fly evolution in order to both increase the immune response’s immediate efficacy and protect it from resistance development. However, theoretical modeling proved by antibiotic selective pressure experiments shows that synergetic antibiotic combinations (unlike antagonistic ones) tend to speed up the antibiotic resistance formation instead of preventing it [38, 39]. It should be taken into account, that the compound used in our experiments contains other constituents alongside with these major AMPs that may take a part in the resistance prevention.

Comparison of resistance development under selective pressure of the compound and conventional antibiotics demonstrates doubtless advantage of the compound in respect of the resistance prevention. None bacterial strain tested was able to develop resistance to the compound whereas resistance to the antibiotics was rapidly elevated. Prospects of the indicated and similar natural compounds as a platform for antimicrobial drug discovery look very attractive when they placed to the global context of antibiotic resistance problem under review [13]. To refine the compound prospects we have used four clinical strains characterized by different profiles of antibiotic resistance summarized in Table 1: antibiotic sensitive *E*. *coli* 774.1, antibiotic multiresistant strains *E*. *coli* 863.1, *K*. *pneumoniae* 104.2 and *A*. *baumannii* 882.2.

Antibiotic sensitive *E*. *coli* 774.1 rapidly developed resistance under selective pressure of third generation cephalosporin cefotaxime but not the compound. Third generation cephalosporin resistant strains of *E*. *coli* are one of most frequent forms of antibiotic resistant pathogens, which require urgents measures for their expansion counteraction [13]. Third-generation cephalosporins replacement, when possible, by the compound-based medication would help to slow down the cephalosporins resistance expansion.

An example of *E*. *coli* 863.1 strain demonstrate other probable field of the compound application. The strain is characterized by broad spectrum of antibiotic resistance. Particularly, it is resistant to third generation cephalosporins. Since the strain sensitivity to the cephalosporin cefoperazone was recovered by beta-lactamase inhibitor sulbactam, resistance to this kind of antibiotics may be attributed to the beta-lactamase activity. The strain remains sensitive to another group of beta-lactam antibiotics, carbapenems, which are often considered as antibiotics of last resort for *E*. *coli* treatment [14]. However, it rapidly develops high level of carbapenem resistance under selective pressure of meropenem (Fig 3C) and may become practically untreatable by carbapenems, third generation cephalosporins and many other antibiotics. Use of the compound instead of carbapenems could allow avoiding this dangerous situation.

Antibiotic resistance profile of *K*. *pneumoniae* 104.2 strain is quite similar to the profile of *E*. *coli* 863.1. It belongs to the third generation cephalosporin resistant strains and retains sensitivity to the carbapenems. The carbapenems are the main remaining treatment option for this kind of *K*. *pneumoniae* infections [13]. However, experiments with meropenem demonstrated that it can easily develop high level of carbapenem resistance as well. Carbapenem resistant *K*. *pneumoniae* is considered among most important pathogens [14]. The compound is active against *K*. *pneumoniae* 104.2 strain and can be potentially used as an alternative to carbapenems. It may help to decrease the carbapenem resistant strains expansion and save carbapenems for systemic life-threatening *K*. *pneumoniae* infections.

Prevalence of multidrug resistant strains typical to *A*. *baumannii* puts it in the forefront of the most dangerous pathogens [14]. The strain 882.2 remains sensitive to polymyxin B, however it decrease efficacy in the course of selection (Table 3). Since polymyxin B therapeutic dosage is strongly limited by the antibiotic toxicity, even small increase of resistance would make it practically unusable. The compound looks prospective as alternative treatment of *A*. *baumannii* multidrug-resistant infections both in terms of antibacterial activity and prevention of resistance development.

Thus, the results of the experimental studies demonstrate prospects of naturally occurring AMP complexes in real clinical situations described above as well as in a broader context of drug resistance prevention and fighting of antibiotic resistant bacteria.
